# Integral and Local Methods for the Evaluation of the Hemostasiological Profile in Sheep at Various Stages of Implantation of a Biodegradable Vascular Graft

**DOI:** 10.17691/stm2022.14.5.03

**Published:** 2022-09-29

**Authors:** O.V. Gruzdeva, E.E. Bychkova, T.Yu. Penskaya, A.A. Kuzmina, L.V. Antonova, L.S. Barbarash

**Affiliations:** Associate Professor, Professor of the Russian Academy of Sciences, Head of the Laboratory of Homeostasis Research; Reserch Institute for Complex Issues of Cardiovascular Diseases, 6 Sosnovy Blvd, Kemerovo, 650002, Russia; Research Assistant, Laboratory of Homeostasis Research; Reserch Institute for Complex Issues of Cardiovascular Diseases, 6 Sosnovy Blvd, Kemerovo, 650002, Russia; Junior Researcher, Laboratory of Homeostasis Research; Reserch Institute for Complex Issues of Cardiovascular Diseases, 6 Sosnovy Blvd, Kemerovo, 650002, Russia; Junior Researcher, Laboratory of Homeostasis Research; Reserch Institute for Complex Issues of Cardiovascular Diseases, 6 Sosnovy Blvd, Kemerovo, 650002, Russia; Head of the Laboratory of Cell Technologies; Reserch Institute for Complex Issues of Cardiovascular Diseases, 6 Sosnovy Blvd, Kemerovo, 650002, Russia; Professor, Academician of the Russian Academy of Sciences, Chief Researcher; Reserch Institute for Complex Issues of Cardiovascular Diseases, 6 Sosnovy Blvd, Kemerovo, 650002, Russia

**Keywords:** hemostasiological profile, integral methods, local methods, implantation of a biodegradable vascular graft, prediction of thrombotic risks

## Abstract

**The aim of this study** was to evaluate the efficiency of local and integral methods of the assessment of the hemostasiological profile in sheep at various stages of implantation of a biodegradable vascular graft.

**Materials and Methods:**

The object of the study was the whole blood of sheep collected at the stage of premedication, during the intraoperative period, and in the early postoperative period. Thromboelastography was used to assess the kinetics of clot formation and changes in its viscoelastic properties in whole blood samples. The thrombin generation test was performed in platelet-rich plasma (PRP) and platelet-poor plasma (PPP) with the assessment of quantitative and temporal parameters. The platelet factor 4 concentration in PRP and PPP was measured by the enzyme immunoassay. The functional activity of platelets in PPP was assessed with inductors and without additional stimulation. Prothrombin complex activity, APTT values, thrombin time, fibrinogen concentration, antithrombin III and protein C activity, soluble fibrin monomer complexes, and fibrinolysis were determined in blood plasma.

**Results:**

Multidirectional changes in the hemostasiological profile at various stages of vascular prosthesis implantation have been revealed. On the one hand, it is an increased prothrombogenic status, on the other hand, it is the development of hypocoagulation. Shortening of the *R* (blood coagulation time) and *K* (clot formation time) intervals and an increase in the angle parameter and maximum amplitude on the thromboelastogram in all the studied periods relative to the reference values, a significant increase in platelet factor 4 in PRP and increased platelet aggregation testified in favor of hypercoagulation. However, the quantitative parameters of the thrombin generation test and a number of coagulogram indicators pointed to hypocoagulation in the intraoperative and early postoperative periods.

**Conclusion:**

The comparative analysis of local tests characterizing the state of hemostasis and indicators of integral methods demonstrated the advantages of the latter in assessing thrombotic risks during implantation of vascular grafts. Local tests are not sufficient to assess the dynamics of the coagulation process in real time and are not always sensitive to hypercoagulation. The use of integral methods will help to fill these gaps, make a timely diagnosis of hypercoagulability and minimize the risks associated with the implantation of vascular grafts in future.

## Introduction

Currently, animal models are widely used in preclinical testing of vascular grafts. According to the literature, an ovine model is mostly close to a human one. It is optimal for the evaluation of growth, permeability, endothelialization, as well as post implantation visualization of tissue-engineered vascular small-diameter grafts [1]. At the same time, about 40% of ovine grafts develop obstruction of implanted vessels [2]. The adequate assessment of the hemostasis system would allow timely diagnosing and preventing those high thrombotic risks. In this concern, the choice of the laboratory test that most accurately detects changes in the hemostatic profile is of importance.

Both local and integral methods are used for the evaluation of the hemostatic potential during surgical intervention. The local methods are aggregatogram, the international normalized ratio (INR), Quick’s prothrombin time, activated partial thromboplastin time (APTT), thrombin time, fibrinogen, antithrombin III, protein C, and many others. Unfortunately, these methods give most limited information characterizing only separate links of hemostasis which does not fully evaluate the coagulation process. Besides, local tests have poor sensitivity to hypercoagulation [3]. It becomes the ground for the application of integral methods which are aimed at the simulation and reflection of the main physiological aspects of hemostasis *in vitro*. They are thromboelastography (TEG) and a thrombin generation test (TGT). TEG is a sensitive diagnostic method that gives the idea of the kinetics of clot formation from primary threads of fibrin to fibrinolysis in the whole blood samples [4]. TGT makes it possible to evaluate the dynamics of formation and inactivation of the key enzyme of hemostasis — thrombin, the synthesis of which takes place in blood plasma as well as on the surface of the activated platelets. Various TGT modifications can be used to evaluate thrombinogenesis associated and not associated with platelets [5].

The study of the platelet factor 4 (PF4) the physiological function of which is blood clotting stimulation is promising in the evaluation of the hemostasis system [6]. Consequently, an increase in PF4 concentration can be regarded as a diagnostic sign of hypercoagulation development. However, to date, there is practically no research work aimed at the study of PF4 changes during surgical intervention.

The modern literature has little data on changes in the ovine hemostasis system during implantation of vascular grafts, and comparison of the results of integral and local methods obtained during this kind of surgery.

**The aim of the study** was to evaluate the efficiency of local and integral methods of assessment of hemostasiological profile in sheep at various stages of implantation of a biodegradable vascular graft.

## Materials and Methods

The study was carried out at the base of the Research Institute for Complex Issues of Cardiovascular Diseases. The study design was approved by the local ethical committee. The work with animals was guided by the International Guiding Principles for Biomedical Research Involving Animals (CIOMS and ICLAS, 2012), Guide for the Care and Use of Laboratory Animals (National Research Council, 2011), as well as ethical principles of the European Convention for the Protection of Vertebrate Animals Used for Experimental and Other Scientific Purposes (Strasbourg, 2006).

The object of the study was the whole blood of Edilbay sheep non-operated before (n=56), with body mass of 42–45 kg. The blood was collected at the stage of premedication (prior to surgery), in the intraoperative period (2 min after unfractionated heparin introduction), and in the early postoperative period (immediately after surgery).

### Description of the surgical intervention

Under anesthesia with isoflurane (5% vol. for induction anesthesia, 2–3% vol. for maintenance anesthesia), artificial lung ventilation was performed with the MinorVet apparatus (Zoomed, China) in the synchronized intermittent mandatory ventilation (SIMV) mode with 100% oxygen inhalation. After treatment and limitation of the operational field, an incision was made on the left in the projection of the sternocleidomastoid muscle between the jugular vein and trachea thus making an access to the carotid artery. The minor branches of the jugular vein and the carotid artery were legated and stripped. The carotid artery was thoroughly separated from the vagosympathetic trunk sharply and a 7–8-cm long artery segment was completely isolated. Prior to clamping the artery, systemic heparinization was performed by intravenous introduction of 5000 units of heparin as well as washing of the inner surface of the graft with the animal blood and subsequent washing of the clearance of the vascular graft from blood with saline solution containing heparin. Artery clamping was performed with atraumatic clamps. After clamping, at a distance of about 6–7 cm between the clamps, a 4-cm long portion of the artery was excised under the angle of 45–60°. Then, a biodegradable vascular graft of the PHBV/PCL/GFmix/heparin/iloprost type with the 4-mm diameter and 4-cm long (1 animal — 1 graft) was sewn into the animal. A continuous suture was performed with Prolene 6-0 thread by knotty sutures or a “parachute” technique. First, a proximal anastomosis was made, the carotid artery was unclamped thus initiating blood flow through the graft to check the quality of anastomosis. Then, the artery was clamped again prior to proximal anastomosis, and distal anastomosis was formed according to the technique given above. Clamping the graft during the operation was not made. Blood flow was initiated through the whole graft after forming distal anastomosis. After that, the wound was sutured layer by layer: deep fascia with subcutaneous cellular tissue was sutured with Vicril 3-0 thread. The skin was sutured with a stapler. After the operation, the permeability of the carotid artery was tested by ultrasound. Regardless of the angiographic picture, the wound was sutured. The average duration of surgery was 1 h 20 min.

### Assessment of hemostasiologic parameters

TEG was performed on the TEG 5000 Thrombelastograf analyzer (Haemonetics, USA). The following parameters were assessed: blood coagulation time (*R* (min)), clot formation time (*K* (min)), maximum amplitude (mm) and angle (°).

The thrombin generation test was performed in the platelet-rich plasma (PRP) and platelet-poor plasma (PPP) with the use of the Technothrombin TGA set on the CEVERON-Alpha analyzer (Technoclone GmbH, Austria). On the TGT curve, the following parameters were assessed: lag time (tLag (min)), peak thrombin (Peak (nmol/L)), time to peak (tPeak (min)), and endogenous thrombin potential (ETP (nmol/min)) — an area under the thrombin generation curve.

PF4 concentrations in PRP and PPP were determined with the help of the enzyme immunoassay using the CUSABio set (China).

To evaluate the platelet functional activity, maximum aggregation in PPP was measured on the Helena AggRAM aggregometer (BioSciences Europe, Great Britain) using the following inductors: adenosindiphosphate (ADP) — 1.25 and 2.5 μg/ml, epinephrine (5 μg/ml), collagen (100 μg/ml) and without additional stimulation.

Quick’s prothrombin time and the INR were determined with the use of ACL 7000-1 coagulometer (Instrumentation Laboratory, USA); APTT, Klaus fibrinogen, thrombin time, protein C, antithrombin III were determined on the Ceveron-alpha analyzer according to the brand guidelines. Fibrinolitic activity of the blood plasma was measured by detecting soluble fibrin monomer complexes in an orthophenanthroline probe (a tablet variant) and XIIa-kallicrein-dependent fibrinolisis test according to the manufacturer’s instruction (Tehnologia-Standart, Russia).

***The statistical processing*** of the results was carried out with the use of the STATISTICA 10.0 software package. The Kholmogorov–Smirnov test was used to evaluate the character of distribution in aggregate on sample data. The results were presented as the median and 25^_th_^ and 75^_th_^ quartile values — Me [Q1; Q3]. The intragroup comparison of the dependent groups with the distribution of the signs different from normal was performed using the Friedman test, paired comparison — using the Wilcoxon test. When comparing three dependent groups, р≤0.017 was taken as the critical level of significance using the Bonferroni correction. Two dependent groups were compared using the Mann–Whitney U test. Statistically significant differences were determined at p<0.05.

## Results

No statistically significant differences in *R*, *К*, or angle values which determine the dynamics of blood coagulation were found at the assessment of thrombus formation during the studied periods (Table 1).

**Table 1:** Dynamics of the thromboelastography parameters in sheep, Me [Q1; Q3]

Parameter	Before surgery (1)	Intraoperative period (2)	Early postoperative period (3)	Intragroup comparison	Paired comparison
*R* (min)	5.0 [4.4; 5.5]	7.6 [5.6; 9.4]	8.0 [6.4; 9.0]	р=0.02	p_1–2_=0.70 p_1–3_=0.01 p_2–3_=0.20
*К* (min)	1.7 [1.3; 1.8]	2.2 [1.6; 2.4]	1.7 [1.4; 2.2]	р=0.40	p_1–2_=0.70 p_1–3_=0.90 p_2–3_=0.70
Angle (°)	66.6 [63.5; 70.7]	64.2 [61.6; 69.7]	67.5 [58.9; 72.6]	р=0.70	p_1–2_=0.80 p_1–3_=0.90 p_2–3_=0.80
Maximum amplitude (mm)	82.6 [80.9; 83.2]	79.8 [78.9; 81.4]	78.3 [75.9; 80.5]	р=0.01	p_1–2_=0.70 p_1–3_=0.01 p_2–3_=0.20

The value of maximum amplitude that characterizes density as a property of a formed clot decreased in the sheep in the early postoperative period by 11% in comparison with the analogous value before the operation. No statistically significant differences were observed in other studied periods.

The contribution of plasma coagulation and platelet links of hemostasis into thrombogenesis was assessed with the TGT in PPP. When PRP was used, the impact of platelets on temporal and quantitative TGT indicators was excluded.

The lag time in PPP increased 6.8 times in the intraoperative period relative to the preoperative values and decreased in the early postoperative period reaching the values before the operation (Table 2).

**Table 2:** Dynamics of the parameters of the thrombin generation test in platelet-rich plasma in sheep, Me [Q1; Q3]

Parameter	Before surgery (1)	Intraoperative period (2)	Early postoperative period (3)	Intragroup comparison	Paired comparison
tLag (min)	4.4 [3.8; 4.5]	29.7 [16.9; 45.8]	5.9 [3.7; 6.7]	р=0.010	р_1–2=_0.017 p_1–3_=0.10 p_2–3_=0.016
tPeak (min)	10.0 [7.8; 10.3]	31.5 [18.4; 47.4]	16.1 [13.3; 24.7]	р=0.030	p_1–2_=0.020 p_1–3_=0.10 p_2–3_=0.020
Peak (nmol/L)	291.5 [197.9; 421.2]	1.8 [1.3; 2.4]	5.9 [2.1; 12.6]	р=0.002	p_1–2_=0.016 p_1–3_=0.016 p_2–3_=0.10
Endogenous thrombin potential (nmol/min)	3360.2 [2912.5; 4014.0]	3.7 [3.2; 11.0]	116.4 [20.0; 333.3]	р=0.001	p_1–2_=0.015 p_1–3_=0.015 p_2–3_=0.016

The peak thrombin concentration in PPP sharply decreased in the intraoperative period relative to the preoperative values and stayed low in the early postoperative period. ETP was practically absent in the intraoperative period in comparison with the data before surgery; it increased in the early postoperative period but did not reach the preoperative values (see Table 2).

In the intraoperative period, tLag and tPeak in PRP increased 5.0 and 2.6 times, respectively, compared to the analogous values before the operation. In contrast to PPP, lag time in PRP remained at a high level in the early postoperative period relative to the preoperative values (Table 3).

**Table 3:** Dynamics of the parameters of the thrombin generation in platelet-poor plasma in sheep, Me [Q1; Q3]

Parameter	Before surgery (1)	Intraoperative period (2)	Early postoperative period (3)	Intragroup comparison	Paired comparison
tLag (min)	5.5 [3.5; 5.7]	27.4 [24.3; 32.6]	13.7 [9.6; 25.9]	р=0.002	p_1–2_=0.01 p_1–3_=0.01 p_2–3_=0.70
tPeak (min)	11.7 [7.4; 14.5]	30.4 [27.0; 41.1]	20.2 [14.5; 29.0]	р=0.001	p_1–2_=0.01 p_1–3_=0.01 p_2–3_=0.07
Peak (nmol/L)	160.1 [102.6; 307.7]	2.2 [1.4; 3.4]	1.6 [1.5; 2.1]	р=0.002	p_1–2_=0.01 p_1–3_=0.01 p_2–3_=0.70
Endogenous thrombin potential (nmol/min)	2773.5 [2248.5; 3261.3]	6.4 [3.9; 9.1]	9.7 [5.9; 18.6]	р=0.001	p_1–2_=0.01 p_1–3_=0.01 p_2–3_=0.30

The Peak parameter in PRP was characterized by the same dynamics as in PPP. ETP was practically absent compared to the preoperative values in the intraoperative and early postoperative period (see Table 3).

**Figure 1.**
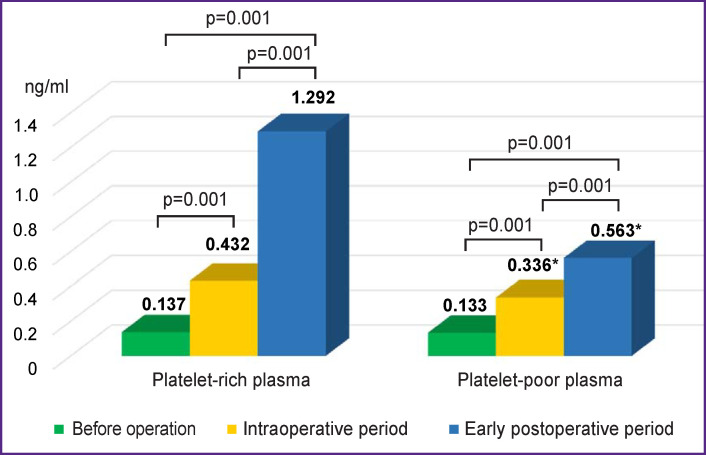
Dynamics of platelet factor 4 in sheep * Statistically significant differences between platelet-rich and platelet-poor plasma, p<0.05

A significant increase in PF4 was found in PPP: 3.2 times in the intraoperative period and 9.4 times in the early postoperative period compared to the data before the operation. A less marked PF4 growth was noted in PRP in the studied periods. PF4 in PRP was 2.5 times higher in the intraoperative period and 4.2 times higher in the early postoperative period compared to the preoperative data (Figure 1). The trend in changes in the dynamics of PF4 in PRP and PPP was the same at all stages of the implantation.

The evaluation of the aggregatogram revealed changes in the functional activity of platelets in sheep at various stages of surgical intervention. In the intraoperative period, higher aggregation compared to preoperative values was typical for animal platelets under the action of inductors and without additional stimulation. In the early postoperative period, the level of maximum platelet aggregation induced by ADP independent on concentration remained at a high level relative to the preoperative values. No statistically significant differences in the parameters of ADP-induced platelet aggregation were observed between the intraoperative and early postoperative periods. Collagen-induced platelet aggregation in the early postoperative period decreased to the preoperative values (Table 4).

**Table 4:** Degree of maximum platelet aggregation (max (%)) in sheep at various stages of surgical intervention, Me [Q1; Q3]

Parameter	Before surgery (1)	Intraoperative period (2)	Early postoperative period (3)	Intragroup comparison	Paired comparison
ADP 1.25 μg/ml	74.8 [72.2; 81.9]	85.4 [83.4; 93.9]	84.5 [82.0; 91.3]	р=0.001	p_1–2_=0.001 p_1–3_=0.001 p_2–3_=0.120
ADP 2.5 μg/ml	83.3 [79.5; 86.4]	89.6 [83.1; 94.1]	87.2 [82.7; 92.5]	р=0.001	p_1–2_=0.001 p_1–3_=0.003 p_2–3_=0.190
Collagen	81.5 [78.3; 85.1]	84.6 [83.1; 89.4]	82.4 [80.3; 87.7]	р=0.001	p_1–2_=0.001 p_1–3_=0.380 p_2–3_=0.001
Epinephrine	1.6 [1.1; 2.4]	13.3 [5.9; 15.2]	15.9 [9.3; 20.2]	р=0.001	p_1–2_=0.001 p_1–3_=0.001 p_2–3_=0.001
Spontaneous aggregation of thrombocytes	3.0 [2.0; 4.0]	4.8 [4.0; 6.0]	4.0 [3.0; 5.0]	р=0.001	p_1–2_=0.003 p_1–3_=0.007 p_2–3_=0.007

The level of maximum epinephrine-induced platelet aggregation continued to increase in the early postoperative period exceeding 9.6 times the preoperative values, 1.2 times the values of the intraoperative period. Spontaneous platelet aggregation decreased a little in the early postoperative period, it was 1.3 times above the preoperative values (see Table 4).

The analysis of the coagulogram data showed that the intraoperative period in the sheep was characterized by a 2.4 times prothrombin decrease and 1.9 times INR increase relative to the analogous preoperative values. In the early postoperative period, a statistically significant increase in prothrombin activity in ovine plasma by 1.6 times and the INR decrease by 1.5 times were observed relative to the values of the intraoperative period (Table 5). The prothrombin complex activity in the early postoperative period did not reach the preoperative values.

**Table 5:** Dynamics of the coagulogram parameters in sheep, Me [Q1; Q3]

Parameter	Before surgery (1)	Intraoperative period (2)	Early postoperative period (3)	Intragroup comparison	Paired comparison
Prothrombin (%)	85.4 [75.2; 108.5]	39.9 [26.6; 51.0]	61.7 [50.0; 68.5]	р=0.001	p_1–2_=0.001 p_1–3_=0.001 p_2–3_=0.010
INR	1.14 [1.0; 1.22]	1.97 [1.57; 2.29]	1.37 [1.29; 1.47]	р=0.001	p_1–2_=0.001 p_1–3_=0.001 p_2–3_=0.001
APTT (s)	26.2 [24.0; 30.5]	188.5 [183.0; 195.0]	186.5 [93.8; 191.0]	р=0.001	p_1–2_=0.001 p_1–3_=0.001 p_2–3_=0.180
Thrombin time (s)	14.1 [12.5; 16.8]	38.5 [36.0; 43.0]	37.5 [32.0; 40.0]	р=0.001	p_1–2_=0.001 p_1–3_=0.001 p_2–3_=0.60
Fibrinogen (g/L)	4.9 [4.2; 6.4]	5.7 [3.7; 8.1]	5.1 [4.0; 6.3]	р=0.10	—

During the intraoperative period, the animal blood plasma showed a 7-fold increase in APTT relative to the preoperative values. In the early postoperative period, the studied parameter remained at the level of the intraoperative period. Similar dynamics in the intraoperative and early postoperative periods was typical for thrombin time. The analysis of fibrinogen concentration in the blood plasma of the sheep did not show statistically significant differences at different stages of surgery.

The thrombus dissolution time increased 1.3 times in the intraoperative period and 1.8 times in the early postoperative period when compared with the same indicator before surgery (Table 6).

**Table 6:** Dynamics of the parameters of the fibrinolytic system in sheep, Me [Q1; Q3]

Parameter	Before surgery (1)	Intraoperative period (2)	Early postoperative period (3)	Intragroup comparison	Paired comparison
Fibrinolysis (min)	6.5 [6.0; 8.0]	8.5 [7.0; 15.0]	12.0 [11.0; 14.0]	р=0.003	p_1–2_=0.001 p_1–3_=0.001 p_2–3_=0.120
Soluble fibrin monomer complexes (mg%)	14.5 [7.5; 16.0]	15.0 [12.0; 21.0]	12.0 [9.0; 15.0]	р=0.001	p_1–2_=0.080 p_1–3_=0.420 p_2–3_=0.001

**Figure 2.**
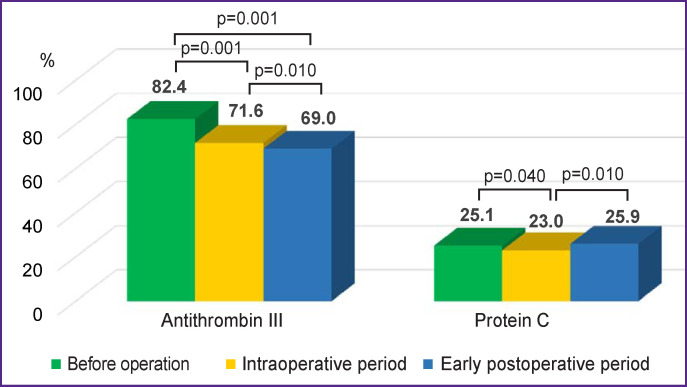
Dynamics of the natural anticoagulant values in sheep

The activity of the natural anticoagulant, antithrombin III, had negative dynamics in the studied periods. It decreased by 11% in the intraoperative period and by 12% in the early postoperative period. The activity of protein C in the blood plasma of the animals decreased in the intraoperative period by 11% relative to the data before surgery; in the early postoperative period, it reached the preoperative values (Figure 2).

## Discussion

Almost any surgical intervention is known to be accompanied by changes in the hemostasis system. However, there is very little data on the focus and severity of these changes during the implantation of vascular grafts in sheep. Besides, the question of the choice of a laboratory method most clearly reflecting changes in the hemostasiological profile during surgery remains open. Two approaches, integral and local ones, were used in our work to evaluate these changes. The use of integral tests makes it possible to characterize the functional state of the hemostasis system and simulate quite accurately the coagulation process *in vitro*, reflecting all three phases of blood coagulation. Integral tests evaluate platelet activity, thrombus formation kinetics, fibrin clot quality, and fibrinolysis, allowing correct interpretation of the pathophysiological mechanisms of coagulation disorders [7]. Local tests for assessing hemostasis are not able to simulate the process of coagulation *in vivo*, despite their widespread use in laboratory practice. They provide data only on the beginning of the coagulation process; therefore, the test result is not representative of the entire clot formation process. The end point of these tests coincides with the initiation phase, giving no information about the phases of strengthening and propagation of the hemostatic system [8]. In addition, local methods do not consider in the least the role of platelets as important participants in blood coagulation processes. Therefore, the results of the local and integral tests do not match, which was found in our work.

The TEG results demonstrated no statistically significant differences in *R* and *K* intervals in the studied periods, despite the introduction of unfractionated heparin during the implantation of vascular grafts. At the same time, local tests (APTT, INR, thrombin time) showed a significant increase in time intervals in the intraoperative and early postoperative periods. The difference in the outcomes may be due to several causes: for example, the use of whole blood in а TEG, and hence the contribution of the platelet hemostasis link, which makes up to 80%, in the formation of a blood clot. Blood plasma is used in local tests. Consequently, laboratory tests model only “fragments” of the blood coagulation process, without giving a full picture of interaction of plasma factors and cellular components of blood. Differences between *R* and APTT, reflecting the hypocoagulant effect of heparin, may also be due to the effect of platelet and endothelial coagulation factors released on activation of the vascular platelet hemostasis link. Heparin production is known to release a tissue factor pathway inhibitor (TFPI) from the vascular endothelium. In the presence of heparin, antithrombin together with TFPI inhibit the activation of blood coagulation. Antithrombin inactivates factor VII associated with tissue factor (TF), while TFPI inhibits the formation of factor VIIa/TF complexes [9]. In addition, animal studies [10] have shown that TFPI itself has antithrombotic properties. However, this effect is leveled at the activation of the platelet link of hemostasis during the implantation of a vascular graft in laboratory animals, which was shown in the thromboelastogram. It was found that the intraoperative period was characterized by an increase in platelet aggregation with induction and without stimulation, which may be associated with the activating effect of heparin directly on platelets and the initiation of platelet adhesion and aggregation during surgery, since in the early postoperative period the maximum degree of collagen-induced and spontaneous aggregation reached preoperative values. In general, the changes observed in the aggregation pattern characterize hypercoagulability and are in line with the TEG data. In addition, the absence of statistically significant changes in the *R* and *K* intervals may indicate a low bioavailability and effectiveness of unfractionated heparin in sheep. An increase in the concentration of PF4 in the intraoperative and early postoperative periods might be a probable explanation for this phenomenon. PF4 is a high-affinity heparin-binding protein that is released during platelet aggregation [11]. The tetrameric shape of PF4 forms a cylindrical structure and includes an equatorial ring. It is composed of positively charged amino acids and forms a binding site for negatively charged heparin. The binding site for heparin and ovine PF4 is located in the C-terminal region of the molecule. The formation of the PF4/heparin complex leads to inactivation of the anticoagulant effect of heparin [12]. Probably, the PF4 effect causes early thrombotic occlusion of 40% of grafts implanted in sheep in the early postoperative period, despite ongoing heparin therapy.

The angle values, which reflect an increase in clot strength and characterize fibrinogen functional activity in whole blood, did not have statistically significant differences in the studied periods. The data obtained are consistent with the content of fibrinogen in the blood plasma, the level of which exceeded the values of the reference interval during the entire observation period. The clot density in the early postoperative period decreased by 11% compared to that before surgery. The maximum amplitude characterizes the state of fibrinogen and platelets. The decrease in clot density is likely to be caused by a statistically significant decrease in the number of platelets in the early postoperative period. It should be noted that in all the studied periods, there was a shortening of the *R* and *K* intervals and an increase in the angle and maximum amplitude relative to the reference values, which indicates an increase in the prothrombogenic status. Consequently, the process of thrombus formation in the ovine blood is faster with the formation of a strong clot at all stages of implantation of vascular grafts.

The thrombin generation test belongs to the integral methods for assessing hemostasis, though differs significantly from TEG. The first difference is associated with the material used for the study: blood plasma is used in TGT, whole blood is used in TEG. The second difference is the principle of the method. TGT is based on the study of the kinetics of thrombin generation by determining the rate of hydrolysis of a thrombin-specific fluorogenic substrate. TEG refers to one of the most direct methods characterizing the functional state of the hemostatic potential and its final result — the formation of a fibrin thrombus, which makes it possible to evaluate the formation of a clot and platelet aggregation simultaneously. These differences can lead to incomparability of the TEG and TGT results.

According to TGT, Peak and ETP before implantation in the PRP and PPP exceeded the values of the reference intervals. Consequently, according to the results of TGT, the prothrombogenic status of the ovine blood before surgery increased, which is consistent with the TEG data. However, due to systemic heparinization, the intensity of unbound and platelet-related thrombinogenesis decreased. This is determined by a decrease in the endogenous thrombin potential and the maximum concentration of thrombin, which indicates the development of hypocoagulation. In the early postoperative period, the endogenous thrombin potential increased in PRP, but did not reach the preoperative values; in PPP it remained at the level of the intraoperative period. The pronounced differences are probably due to the contribution of the platelet link of hemostasis to thrombinogenesis occurring in PRP, in contrast to PPP. Platelet activation is accompanied by the translocation of negatively charged phospholipids to the outer surface of their membranes, thus creating conditions for the formation of thrombin from prothrombin [13].

The fibrinolytic activity of the blood plasma in the intraoperative and early postoperative periods decreased (this is indicated by the prolongation of the thrombus dissolution time) but remained within the reference intervals. At the same time, a statistically significant decrease in the level of antithrombin III was observed in the intraoperative and early postoperative periods. Theoretically, the increased use of antithrombin to realize the main effects of heparin during implantation of a vascular graft could contribute to a decrease in its activity. Changes in the functional activity of protein C during the intraoperative period are probably due to the dysfunction of the vascular epithelium during implantation, on the surface of which the protein is activated [14]. A decrease in the activity of natural anticoagulants is traditionally considered as an indicator of hypercoagulability, which is not consistent with the data of the coagulogram and TGT but corresponds to the results of TEG.

Thus, a comparative analysis of the local tests characterizing the state of hemostasis and indicators of integral methods, carried out as part of our study, demonstrated the advantages of the latter in assessing thrombotic risks during implantation of vascular grafts.

## Conclusion

Local tests do not make it possible to assess the dynamics of the coagulation process in real time and are not always sensitive to hypercoagulation. The use of integral methods allows filling these gaps, diagnosing hypercoagulability in a timely manner, and minimizing further the risks associated with the implantation of vascular grafts.
